# Prevalence and prognosis of fulminant type 1 diabetes mellitus in The Middle East: a comparative analysis in a 5-year nationwide cohort

**DOI:** 10.1186/s12902-024-01559-8

**Published:** 2024-03-11

**Authors:** Fateen Ata, Adeel Ahmad Khan, Ibrahim Khamees, Sham AlKodmani, Anas Al-Sadi, Khaled Bani Yaseen, Bassam Muthanna, Angela Godwin, Stephen Frederick Beer, Mohammed Bashir

**Affiliations:** 1 Department of Endocrinology, Hamad Medical Corporation, Doha, Qatar; 2 Department of Internal Medicine, University of Missouri, Kansas City, MO USA; 3 Department of Internal Medicine, Hamad Medical Corporation, Doha, Qatar; 4 Department of Medicine, Louis A. Weiss Memorial Hospital, Chicago, USA; 5 Department of Laboratory Medicine, Hamad Medical Corporation, Doha, Qatar

**Keywords:** Diabetic ketoacidosis, DKA, Classic Type 1 diabetes, Fulminant type 1 diabetes, Diabetes mellitus

## Abstract

**Purpose:**

To analyze the prevalence and progression of fulminant type 1 diabetes (FT1D) in Qatar.

**Methods:**

This retrospective study analyzed consecutive index- diabetic ketoacidosis (DKA) admissions (2015–2020) among patients with new-onset T1D (NT1D) in Qatar.

**Results:**

Of the 242 patients, 2.5% fulfilled the FT1D diagnostic criteria. FT1D patients were younger (median-age 4-years vs.15-years in classic-T1D). Gender distribution in FT1D was equal, whereas the classic-T1D group showed a female predominance at 57.6% (*n* = 136). FT1D patients had a mean C-peptide of 0.11 ± 0.09 ng/ml, compared to 0.53 ± 0.45 ng/ml in classic-T1D. FT1D patients had a median length of stay (LOS) of 1 day (1-2.2) and a DKA duration of 11.25 h (11–15). The median (length of stay) LOS and DKA duration in classic-T1D patients were 2.5 days (1-3.9) and 15.4 h (11–23), respectively. The FT1D subset primarily consisted of moderate (83.3%) and severe 916.7%) DKA, whereas classic T1D had 25.4% mild, 60.6% moderate, and 14% severe DKA cases. FT1D was associated with a higher median white cell count (22.3 × 10^3^/uL) at admission compared to classic T1D (10.6 × 10^3^/uL). ICU admission was needed for 66.6% of FT1D patients, compared to 38.1% of classic-T1D patients. None of the patients in the FT1D group had mortality, while two died in the classic-T1D group.

**Conclusion:**

This is the first study establishing the existence of FT1D in ME, which presented distinctively from classic-T1D, exhibiting earlier age onset and higher critical care requirements. However, the clinical outcomes in patients with FT1D seem similar to classic T1D.

## Introduction

Fulminant type 1 diabetes (FT1D) is the most recently recognized subtype of diabetes mellitus (DM), with its first description in the early 2000s by Imagawa et al. [1]. FT1D manifests with rapid onset ketoacidosis within days of development of hyperglycemia symptoms, pronounced serum glucose in juxtaposition with modest glycated hemoglobin (HBA1C) levels, the near-complete destruction of islet B cells at the onset of disease evident by very low C-peptide levels [1–3]. This is usually but not always accompanied by gastrointestinal or respiratory symptoms preceding the hyperglycemic episode, negative islet antibodies, and an increased incidence of acute complications such as severe hypoglycemia and serious metabolic derangements [1–3]. Some studies have also found elevated amylase and lipase levels in FT1D patients, suggesting potential future inclusion in its diagnostic criteria [4]. Gastrointestinal symptoms upon presentation, in conjunction with elevated levels of amylase and lipase, suggest a more comprehensive pancreatic involvement, extending beyond just the endocrine cells [5]. The etiology of FT1D is unclear; however, various studies link the onset of FT1D after immune checkpoint inhibitor treatment for malignancies [1]. Certain viral infections and the subsequent immune response are also thought to trigger the development of FT1D [4].

Prognostically, FT1D’s unanticipated and aggressive clinical course at onset distinguishes it from classic T1D, often signifying a more dire prognosis [4]. However, data concerning differences in short-term and long-term outcomes of patients is lacking, and a detailed assessment of its prognosis is yet to be ascertained [6]. Beta cells usually possess the ability to regenerate to some extent, which is seen in classic T1D. A profound lacuna exists in discerning the intricate distinctions between FT1D and classic T1D, advocating for further clinical explorations. Moreover, it is yet to be determined if the distinct presenting features between FT1D and classic T1D translate into divergent clinical outcomes concerning both immediate and long-term diabetic complications.

Owing to its infrequent nature and limited studies, the true prevalence of FT1D remains elusive, with notable geographic variations. Subsequently, FT1D has struggled to be accepted as a separate subtype of T1D globally, with the World Health Organization (WHO) classifying it as a distinct subcategory of T1D but not the American Diabetes Association (ADA) [7]. The clearly distinct clinical and demographic features of FT1D from classic T1D translating into a difference in prognosis or progression of T1D would support its wide recognition; however, the results from studies have been limited and variable. Different outcomes might be more expected during the initial DKA admissions and not in the long-term complications of T1D, as eventually, all patients with T1D, regardless of subtypes, are entirely dependent on external insulin with complete destruction of beta cells over time. In a nationwide study from Japan, Imagawa et al. reported worse presenting features, such as more loss of consciousness and more severe electrolyte disturbances in FT1D compared to classic T1D [7]. The Japanese study reported a 20% point prevalence of FT1D among all T1D with new DKA. Another study from Korea reported a 7.1% point prevalence of FT1D among all newly diagnosed T1D patients. The study also reported worse metabolic derangements in the FT1D group [8]. Similarly, variable prevalence has been reported in studies from China [9]. It is important to note that authors used different populations to establish prevalence, such as all patients with new ketosis and diabetes, all new T1D cases regardless of ketosis, and those with new ketosis and T1D.

A knowledge void surrounds the incidence of FT1D within the Middle Eastern region and is limited to case reports. The incidence of T1D in the Arab population has been reported to range from 2.5 to 29/100,000 patients [10]. Qatar registers a considerably higher T1D incidence at 33.2 per 100,000 patients—ranking fourth among 89 nations studied [11]. Childhood T1D incidence has witnessed an upsurge in Qatar, with data from 2016 reporting 23.64/100,000 patients—significantly surpassing Japan’s 2.25/100,000 patients during the same time [12, 13]. However, it is unknown if similar disparities exist in the prevalence of FT1D.

The prevalence of FT1D in the Middle Eastern and Asian populations, possible triggers, the response of these patients to insulin therapy, and the risks of severe hypoglycemia and metabolic derangements, including DKA, remain largely unexplored. We aimed to conduct a retrospective study to evaluate the prevalence of FT1D and its clinical course compared to classic T1D in Qatar, home to a predominantly South Asian and Middle Eastern Arabic population.

## Materials and methods

### Study design

We conducted a retrospective study on all consecutive patients with new T1D admitted with index DKA from January 2015 to August 2021 in four public hospitals in Qatar. These were the only public hospitals in Qatar with medical emergency services receiving patients at the time of study.

### Ethical approval

Ethical approval for this study was sought from the institutional review board at the Medical Research Centre (MRC), Qatar (MRC-01-21-698). The need for Informed consent was waived as this was a retrospective data review.

### Definitions and inclusion criteria

The inclusion criteria were patients of all ages with a new diagnosis of T1D on the index DKA admission. The diagnosis of T1D was available in the electronic medical records (EMR). It was manually cross-checked based on glycated hemoglobin (HbA1c) ≥ 6.5% or a fasting glucose ≥ 7.0 mmol/L at the time of DKA diagnosis, low C-peptide levels where available, and positive anti-glutamic acid decarboxylase (GAD) antibodies where available. The ketoacidosis diagnosis was defined using the American diabetes association DKA diagnostic criteria: pH < 7.3, serum bicarbonate ≤ 18 meq/L, anion gap > 10 mmol/L, and ketonemia/ketonuria [14]. DKA was categorized into mild, moderate or severe per the ADA DKA diagnostic criteria (using PH, bicarbonate, and anion gap to categorize the severity) [14]. We did not define the glucose level to diagnose DKA in order not to miss euglycemic DKA. We excluded patients with other causes of ketoacidosis, such as starvation and alcohol-induced ketoacidosis. FT1D was defined as new-onset T1D in patients presenting with DKA for the first time, with HBA1C level < 8.7%, glucose > 16 mmol/l, serum C-peptide < 0.3 ng/ml, with onset of hyperglycaemic symptoms in the last seven days before admission or ketonemia/ketonuria at initial presentation [15]. DKA resolution was defined as the closure of the anion gap. Serum ketones were monitored in many cases to document resolution, although this was not uniformly possible for all patients due to varying availability. As anion gap closure was documented in all included patients, hence it was used as the marker of resolution. Classic T1D was defined as new-onset T1D presenting with DKA with C-peptide levels ≥ 0.3 ng/ml, HBA1C ≥ 8.7%, regardless of initial serum glucose levels and hyperglycaemic symptoms’ duration before progressing to DKA. The criteria for ICU admission were determined in accordance with the hospital corporation’s DKA treatment guidelines, which align with international recommendations [16]. Admission to the ICU was considered under the following conditions: persistent hypotension or oliguria despite the administration of 2 L of normal saline; mental obtundation; pregnancy; septic shock; a pH level below 7.15; or heart failure. It is important to note that these criteria were supplemented by the clinical judgment of the attending physician, allowing for individualized patient care decisions.

Firstly, we identified 1949 patients with index DKA admissions between 2015 and 2021 to four hospitals of Hamad Medical Corporation, Qatar (Hamad General Hospital, Al-Khor Hospital, Al-Wakra Hospital, and Hazam Mebaireek General Hospital) through EMR. Patients with pre-existing diabetes (T1D or T2D) were then excluded, followed by the exclusion of newly diagnosed T2D (diagnosed at the studied admission). The subsequent cohort consisted of 259 patients with a new diagnosis of T1D established on the index DKA admission. Of these, 17 patients did not fulfill the American diabetes association DKA criteria and were removed, leaving a final cohort of 242 patients.

### Data collection

EMR was used to extract the required data from the finalized cohort. Based on the definitions, patients were categorized into FT1D or classic T1D. Variables included patients’ age, sex, body mass index (BMI), ethnicity, comorbidities, and presenting symptoms. Laboratory data on admission included the serum glucose, HBA1C, serum and/or urine ketone levels, pH, lactate, bicarbonate, sodium, potassium, chloride, anion gap, creatinine clearance, lipid profile, liver function tests, serum amylase, serum lipase, C-peptide level, anti- GAD antibody levels. Outcome data included length of hospital stay in days, DKA duration in hours, admission to intensive care unit (ICU), ICU stay in days, DKA recurrence at 3 and 6 months, 90-day all-cause readmission, and in-hospital mortality.

Commonly available markers of T1D progression, including HBA1c levels, creatinine clearance, development of DM retinopathy, and nephropathy, were included in follow-up data. The follow-up data was collected until August 2021, the pre-specified study period per the IRB-approved protocol. Management of DKA across all facilities within Hamad Medical Corporation adheres to a uniform protocol based on the ADA management guidelines. Given the anticipated uniformity in treatment approaches, hospital-specific treatment variables were not analyzed.

### Biochemical assays

The assays for key biomarkers were done at the Department of Laboratory Medicine and Pathology, Hamad Medical Corporation, which is accredited by the College of American Pathologists (CAP). Tests were performed as per manufacturer's recommended protocol. C-peptide levels were measured using an electrochemiluminescence immunoassay. HbA1c percentages were determined through a turbidimetric inhibition immunoassay. Bicarbonate levels were measured enzymatically using phosphoenol pyruvate carboxylase (PEPC). A kinetic enzymatic assay was used for β-hydroxybutyrate. The coefficient of variation for these tests during the study period was 3.27% for C- peptide at a mean value of 5.00ng/mL, 3.64% for HbA1c at a mean value of 5.17%, 6.81% for bicarbonate at a mean value of 20.04 mmol/L and 8.29% at a mean value of 0.25 mmol/L for β-hydroxybutyrate.

### Study outcomes

The primary outcome of our study was the prevalence of FT1D among all newly diagnosed T1D patients presenting with DKA. Secondary outcomes included severity of DKA, hospital length of stay, ICU admission rates, ICU length of stay, and DKA duration in hours. Additional secondary outcomes were HBA1C, creatinine clearance, and diabetes-related retinopathy development at follow-up.

### Statistical analyses

We used descriptive statistics to present the demographic data of the study cohort. The cohort was categorized into FT1D and Classic T1D based on predefined criteria. We summarised continuous variables as mean (SD) and median (IQR) depending upon the normality of data and categorical variables as percentages. Due to the anticipated low prevalence of FT1D, we did not perform formal statistical tests. All analysis was performed on STATA 15.

## Results

### Clinical characteristics of the study cohort

The baseline characteristics of the cohort are presented in Table 1. The median age of the study cohort was 15 years (7–25), out of which 139 (57.4%) were females. The median body mass index (BMI) was 20 kg/m^2^ (15.6–24.4). The median random blood glucose at admission was 26.4 mmol/L (20-31.7), and the median glycated hemoglobin (HBA1C) was 11.7 (10.6–13.3). A family history of T1D was reported in 25.2% (29) of patients. Among the study cohort, 43% (104) patients required intensive care unit (ICU) admission, with a median length of hospital stay at two days (1–2). The median hospital length of stay was 2.4 days (1-3.9) for all patients, with a median DKA duration of 15 h (11–23). Only two patients (0.8%) died during the hospitalization.

**Table 1:** Baseline Characteristics of all patients with New-onset T1D admitted with Index DKA episode

Baseline characteristics	Results (*N* = 242)
**Age**, Years	15 (7–25)
**Gender**	
Male Female	139 (57.4%) 103 (42.6%)
**Comorbidities**	
Dyslipidaemia Coronary artery disease Hypertension	3 (1.24%) 2 (0.8%) 4 (1.6%)
**Diabetes type**	
Classic Fulminant	236 (97.5%) 6 (2.5%)
**BMI**	20 (15.6–24.4)
**Glucose at admission** mmol/L	26.4 (20-31.7)
**HbA1c** at admission (< 6.5%)	11.7 (10.6–13.3)
**Family History of T1D**	29 (25.2%)
**Need for admission to ICU**	104 (43.1%)
**ICU length of stay**, Days	2 (1–2)
**DKA duration**, Hours	15 (11–23)
**Hospital length of stay**, Days	2.4 (1-3.9)
**In-hospital mortality**	2 (0.8%)

Data presented as Mean ± SD, Median (IQR), and Numbers (%) as appropriate. (T1D: Type 1 diabetes mellitus, IQR: Interquartile range, SD: Standard deviation, BMI: Body mass index, HBA1c: Glycated hemoglobin, DKA: Diabetic ketoacidosis, ICU: Intensive care unit)

### Clinical characteristics of FT1D and classic T1D subgroups

Of the 242 patients newly diagnosed with T1D on the index DKA admission, six fulfilled the FT1D criteria, establishing a point prevalence at 2.5% among all new T1D cases presenting with DKA. The baseline characteristics, evolution of DKA, and follow-up data of FT1D and classic T1D are detailed in Table 2. The median age of patients with FT1D was four years (IQR 3–16) compared to 15 years (IQR 7–25) in patients with classic T1D. Gender distribution was equal (50% each) in patients with FT1D, whereas 57.6% (136) of patients diagnosed with classic T1D were females. Five patients with FT1D (83.3%) were Arabs, and one (16.7%) was Asian. On the other hand, 167 (70.7%) patients with classic T1D were Arabs, and 42 (17.7%) were Asians. The median BMI in the FT1D group and classic T1D group was 17.7 kg/m^2^ (16.4–19.9) and 20.1 kg/m^2^ (15.6–24.5). The mean C-peptide level in the FT1D group was 0.11 ± 0.09 ng/ml compared to 0.53 ± 0.45 ng/ml in the classic T1D group. The median creatinine clearance at baseline in the FT1D group was 52.7 ml/min (43-80.1) compared to 87.2 (63.8-126.6) ml/min in the classic T1D group. The median hospital length of stay in patients with FT1D and classic T1D group was one day (1-2.2) and 2.5 days (1-3.9), respectively. Similarly, the median DKA duration in the FT1D and classic T1D groups was 11.25 h (11–15) and 15.4 h (11–23). Most patients in the FT1D group had a moderate DKA (83.3%), whereas 16.7% had severe DKA. Conversely, in the classic T1D group, 25.4% had mild, 60.6% had moderate, and 14% had severe DKA episodes. Most FT1D patients (66.6%) were admitted to ICU, whereas only 38.1% of classical T1DM patients were admitted to ICU. There was no mortality in the FT1D group, compared to two (0.8%) in-hospital mortalities in the classic T1D group.

**Table 2:** Clinical Characteristics of Patients with New-onset Fulminant T1D and Classic T1D admitted with Index DKA episode

Baseline characteristics	Fulminant T1D (*N* = 6)	Classic T1D (*N* = 236)
Age, years	4 (3–16)	15 (7–25)
**Gender**		
Male Female	3 (50%) 3 (50%)	136 (57.6%) 100 (42.2%)
**Ethnicity**		
Arabs Asian Others	5 (83.3%%) 1 (16.7%) 0	167 (70.7%) 42 (17.7%) 27 (11.4%)
BMI, kg/m^2^	17.7 (16.4–19.9)	20.1 (15.6–24.5)
Anti GAD Ab, IU/mL	160 (18.9–431)	199 (42-1407)
C-peptide (1.1–4.4 ng/ml)	0.11 ± 0.09	0.53 ± 0.45
Glucose at admission, mmol/L	24.7 (21.8–29.6)	26.4 (20-31.7)
HbA1c at admission (< 6.5%)	8.05 (7.5–8.2)	11.8 (10.7–13.3)
Lipase (13–60 U/L)	12 (10–75)	27 (17–70)
Alkaline Phosphatase	300 (189.5-342.5)	246 (132–351)
AST (0–32 U/L)	27.5 (18.5–78)	21 (16–27)
ALT (0–33 U/L)	27.5 (21.5–70)	20 (15–27)
WBC admission (4–10 × 10^3^/uL)	22.3 (12.8–22.7)	10.6 (7.6–14.5)
CrCL at admission (ml/min)	52.7 (43-80.1)	87.2 (63.8-126.6)
BHB at admission (0.03–0.3 mmol/L)	4.5 (4.4–6.7)	6 (5.1–7.2)
Lactate at admission (0.5–2.2 mmol/L)	2.3 ± 0.8	2 ± 2.8
Serum pH at admission	7.11 ± 0.1	7.14 ± 0.12
Bicarbonate at admission (22–29 mmol/L)	7.95 ± 3.4	9.2 ± 4.2
Anion Gap at admission (< 10)	25.6 (23.4–27.2)	24.1 (20-27.7)
Length of stay, Days	1 (1-2.2)	2.5 (1-3.9)
DKA duration, Hours	11.25 (11–15)	15.4 (11–23)
**DKA severity**		
Mild Moderate Severe	0 5 (83.3%) 1 (16.7%)	60 (25.4%) 143 (60.6%) 33 (14%)
In-hospital mortality	0	2 (0.8%)
Need for admission to ICU	4 (66.6%)	90 (38.1%)
LOS in ICU	1.5 ± 0.6	3.6 ± 13.6
**DKA recurrence**		
6-month 12-month	1 (16.7%) 0	15 (6.4%) 12 (5.1%)
90-day readmission	1 (16.7%)	33 (14%)
**Follow up data**		
HBA1C (< 6.5%) CrCL (ml/min) CrCL < 60 DM retinopathy	8.8 (7.2–12.8) 143.6 (124.5-178.5) 0 0	9.7 (8–12) 160.1 (126.7-210.4) 2 (0.8%) 7 (3%)

Data presented as Mean ± SD, Median (IQR), and Numbers (%) as appropriate. (T1D: Type 1 diabetes mellitus, NV: Normal Value, IQR: Interquartile range, SD: Standard deviation, HBA1c: Glycated haemoglobin, Ab: Antibody, DKA: Diabetic ketoacidosis, ICU: Intensive care unit, GAD: Glutamic acid decarboxylase, LOS: Length of stay, CrCl: Creatinine clearance, DM: Diabetes mellitus, BHB: Beta-hydroxybutyrate, AST: Aspartate aminotransferase, ALT: Alanine aminotransferase, WBC: White blood cell count)

### Follow-up details of FT1D and classic T1D

The average follow-up time was four years. Follow-up data is available in Table 2. At follow-up, the median HBA1C in the FT1D and classic T1D groups was 8.8% (7.2–12.8) and 9.7% (8–12), respectively. Median creatinine clearance in the FT1D group was 143.6 ml/min (124.5-178.5), compared to 160.1 ml/min (126.7-210.4) in the classic T1D group. None of the patients in the FT1D group had a creatinine clearance < 60 in the FT1D group, whereas two patients (0.8%) in classic T1D had creatinine clearance < 60 ml/min at follow-up. Similarly, diabetic retinopathy was not reported in any patients with FT1D at follow-up, compared to seven (3%) patients in the classic T1D group.

## Discussion

This study highlights the existence of fulminant type 1 diabetes mellitus (FT1D) in the Middle East. Given that the diagnosis of FT1D is made on the initial presentation with diabetic ketoacidosis, we analyzed all patients presenting with their first DKA episode for five consecutive years (2015–2020). Of all the patients diagnosed with T1D, the point prevalence of FT1D was established at 2.5%. Patients with FT1D showed distinct clinical features when compared to classic T1D. These included a younger median age of onset (4 years vs. 15 years), higher indicators of inflammation such as higher median white cell counts (22.3 × 10^3^ vs. 10.6 × 10^3^), more patients with moderate and severe DKA compared to classic T1D, higher needs for intensive care unit (ICU) admission (66.6% vs. 38.1%) albeit a shorter ICU mean length of stay (LOS) (1.5 days vs. 3.6 days), shorter median ketoacidosis duration (11.25 h vs. 15.4 h), and a shorter hospital median LOS (1 day compared to 2.5 days in classic T1D). None of the patients in the FT1D group experienced in-hospital mortality during the index DKA admission, whereas in classic T1D, two patients died. Clinically different features of FT1D in Qatar distinguished it from classic T1D; however, it does not seem to carry an inferior prognosis compared to classic T1D.

FT1D came into clinical attention by observing a rare trend of certain individuals presenting with rapid onset DKA (with the onset of symptoms within one week), with hyperglycemia in the presence of only mildly elevated glycated hemoglobin (HBA1C), and excessively suppressed C-peptide levels. This indicates a considerably rapid development of hyperglycemia in the body coupled with a near-complete destruction of beta cells [17]. This contradicts classic T1D, which ensues with a latent state with a gradual loss of beta cell function, resulting in a slow and persistent rise in blood glucose levels. Ultimately, patients reach a point where the disease becomes symptomatic clinically, and laboratory investigations correlate with a gradual process of unchecked hyperglycemia via raised glucose coupled with considerably higher HBA1C levels with some beta cell function usually preserved at the time of diagnosis. This aligns with the concept of stage-wise destruction of beta cells prior to clinically significant T1D [18]. HBA1C level at the time of diagnosis of classic T1D is reported to be around 11% [19]. However, this also includes cases diagnosed incidentally and with mild hyperglycaemic symptoms. Patients diagnosed with index DKA admission may have even higher HBA1C levels.

Previous studies have limited data on the comparison of clinical outcomes in patients with FT1D and T1D, such as increased LOS, prolonged duration of DKA, increased ICU care, or higher mortality rates [2]. Our study compared both groups and supported the idea of no clinically meaningful worse acute complications. These patients did require more ICU admissions; the LOS in ICU was shorter, and total LOS was also shorter compared to classic T1D. Although patients with FT1D did show worse inflammatory markers, such as higher WBC count, whether this would result in detrimental short-term outcomes remains a debate and needs further comparative studies with larger sample sizes.

We had notable differences in our cohort compared to most previous studies, such as our patient population with FT1D was notably younger than classic T1D. Previous studies showed FT1D to be much more common in adults than in the pediatric population [7]. Secondly, we did not find FT1D in any pregnant female, contrary to previous studies from East Asian countries [7, 9]. We also did not identify any previously known triggering factors of FT1D such as certain viral infections, COVID-19 infection, or use of immune checkpoint inhibitors [20]. Only one patient in our FT1D cohort had an infectious trigger, but no specific virus was isolated in him. Although this might be due to different sample sizes, it is worth noting that diabetes has significant geographical variation, which may be indicated by a predominantly pediatric FT1D in the Middle East compared to the East Asian cohorts. The class II human leukocyte antigen (HLA) gene is implicated in the genetic driver behind FT1D [17]. As genetic diseases are well-known to have ethnic and geographically variable presentations, FT1D may also carry a different phenotype in the Middle East compared to Japan. Much remains unknown about FT1D due to its low occurrence in most parts of the world. More studies are needed explicitly looking into acute and long-term complications of diabetes among FT1D patients to ascertain its clinical meaningfulness as a separate entity. In our patients identified having FT1D, 67% had positive anti GAD antibodies, one had negative antibody and anti-GAD was not available for one patient. Originally, Imagawa and colleagues recognized that patients with FT1D typically presented with negative anti-GAD antibodies, leading to the initial characterization of FT1D with negative anti-GAD antibodies. Over time, with the emergence of more comprehensive data from various cohorts, patients with FT1D having positive anti-GAD antibodies were also observed. Consequently, the presence of negative anti-GAD antibodies is no longer a mandatory criterion for diagnosing FT1D. This evolution in understanding was elaborated by Imagawa et al. in their 2007 comprehensive review [21].

The strength of our study lies in its novelty, being the first in the region to establish the prevalence of FT1D. The patient cohort (all index DKA admissions with T1D as a new diagnosis) was carefully selected to reflect the most accurate point prevalence of FT1D. We applied the ADA criteria of DKA diagnosis to improve the internal validity of the data. The nationwide inclusion of patients from all the public hospitals receiving most patients with DKA allowed more meaningful insights. However, the results of this study have to be judged against its limitations. Firstly, being retrospective in nature, this study carries a possibility of selection bias and reliance on retrospectively available data. This limited our ability to validate the diagnosis of all the patients as many patients did not have C-peptide and anti-GAD antibody levels done. Additionally, the validity of diagnosis was also limited by unavailability of other antibodies (anti IA-2 and anti ZnT8) in the hospital laboratories. The retrospective design also precluded the collection of data on immediate post-DKA complications such as cerebral edema, infections, and lactic acidosis. We did not have the genetic data of the patients with index DKA admissions who were diagnosed with T1DM, hence limiting the interpretation of our results on molecular level. We did not have enough data on inflammatory markers (such as CRP) apart from WBC to compare FT1D and classic T1D which limited our ability to conclude if one type has a more aggressive course compared to the other. Another limitation of this study is a relatively short average follow-up duration of four years, given that long-term complications associated with T1D are generally not anticipated to occur before a five-year timeframe. The comparatively small number of FT1D patients in our cohort may limit the generalizability of specific findings from the study, emphasizing a need for more extensive multination studies from the Middle East and South Asian countries. Nevertheless, this study opens the door to further research on FT1D in the region, which may play a role in the global acceptability of FT1D in the coming years.

## Conclusion

This is the first study from the region establishing the existence of FT1D among Arabs and South Asians. FT1D in the Middle East presented distinctively from classic T1D, exhibiting earlier age of onset, more ICU admissions, and more pronounced DKA severity. However, the clinical outcomes in patients with FT1D do not seem worse than classic T1D. Further research is needed to understand the clinical meaningfulness of FT1D.

## Data Availability

Data sharing requires permission from the Ministry of Public Health, Qatar. Any request for datasets requestions can be made to the Medical Research Center (MRC) Qatar at Hamad Medical Corporation, which will seek legal permission form the MOPH before data sharing. MRChelpdesk@hamad.qa. The corresponding author Fateen Ata can be contacted at Fata@hamad.qa to initiate data availability request.
